# A re-survey in 2019-2021 of winter bird communities in the Oregon Coast Range, USA, initially surveyed in 1968-1970

**DOI:** 10.3897/BDJ.10.e91511

**Published:** 2022-12-20

**Authors:** Nolan M Clements, W. Douglas Robinson

**Affiliations:** 1 Oregon State University, Corvallis, United States of America Oregon State University Corvallis United States of America

**Keywords:** avian communities, benchmark survey, bird abundance, long-term change, Pacific Northwest

## Abstract

**Background:**

Long-term changes in bird populations during winter are poorly evaluated in many parts of the world. We re-surveyed forest bird communities during winter, 2019-2021, in seven large plots originally surveyed from 1968 through to 1970, near Corvallis, Oregon, USA by Stanley Anderson, a graduate student at Oregon State University in the 1960s. Anderson counted birds and measured forest plant communities within the forests dominated by Douglas-fir (*Pseudotsugamenziesii*) in the Coast Range Mountain foothills. His thesis included plot locations, summaries of vegetation characteristics and point estimates of bird densities for each plot. To our knowledge, the Anderson data represent the oldest structured survey of Pacific Northwest winter forest bird communities with density estimates. Given the paucity of similar data, we re-surveyed his plots after aligning methods with his and adding modern components (distance and time interval sampling) to facilitate comparisons of changes in abundances. We preserved more extensive metadata than were preserved from Anderson's surveys, including georeferenced point count survey locations to facilitate more precisely repeatable future re-surveys.

**New information:**

Original surveys of winter bird populations in the Pacific Northwest, USA, based on georeferenced locations within seven large plots originally surveyed, 1968-1970. In addition to raw count data of all bird species detected, we include information from distance sampling and time-interval sampling methods. To our knowledge, this is one of the only structured surveys of winter forest bird populations in the Coast Range Mountains, USA.

## Introduction

Long-term data that utilise standardised and structured methodologies are ideal for quantifying change in bird populations. With increasing interest in quantifying the drivers of biodiversity responses to climate change and other forms of environmental change, the perspective that comparisons with older datasets provide elevates the importance of historic surveys. In addition, preserving metadata from current surveys so that future re-surveys can evaluate temporal change in statistically rigorous ways also elevates the value of current biodiversity surveys. Unfortunately, long-term survey data do not exist for most biogeographic regions. An alternative method to assess change across time is to compare historic collections of short-term data with modern re-surveys of the same sites. Such re-surveys can provide snapshots of potential changes in bird abundance, occupancy, and community composition (Tingley 2017). Many historical datasets vary in the quality of data and metadata they preserved, which can reduce the strength of inference when comparing historic with modern re-survey results. When modern methodology can be sufficiently aligned with the historic methods used, evaluations of change can be more robust. Modern re-surveys should also anticipate future interest in additional re-surveys, so modern counting methods and preservation of georeferenced count location data become essential (Robinson and Curtis 2020, Robinson et al. 2020). In particular, survey methods that allow precise repeatability of surveys and collect data that may be used to assess variability amongst observers in detections of birds and other detectability parameters are now important.

This paper shares data from re-surveys in 2019-2022 of seven large plots in the Oregon Coast Range Mountains originally surveyed in 1968-1970. Anderson (1970, 1972) surveyed bird abundances and inventoried species, providing point estimates of abundance for all species he encountered within his plots. He used multiple methods, but reported that his point counts formed the primary basis for his abundance estimates. Although his raw data were not preserved, his thesis, uniquely for that era, presented the abundance estimates for each plot and species. To compare with his results, we conducted approximately 320 stationary point counts which resulted in 4613 avian records. Given the rarity of structured winter bird surveys from this region, we share the raw data and metadata to facilitate future re-surveys and provide data for additional regional comparisons of winter bird communities. Our workflow for aligning the two datasets are described in Clements (2022). Comparisons with the original survey data show declines of four of the six most regularly detected forest bird species, one of the six species remaining relatively stable in numbers and one increasing strongly (Clements 2022). Species richness overall was consistent through time.

## Sampling methods

### Sampling description

**Avian surveys**

Anderson (1970, 1972) noted the location of his plots, which were quarter sections (65 ha) within the Township and Range divisions of the Public Land Survey System. The exact locations of Anderson's bird surveys within quarter sections were unclear but most quarter sections were within more extensively forested areas, as determined from aerial photos. Therefore, we conducted our surveys within the same quarter sections and assumed, because we were surveying the same habitats, that our survey results would be reliably comparable to Anderson’s. In two cases (plots 6 and 7), access was reduced because of COVID-19-related regulations so some counts we conducted were outside of the quarter-sections, but within 250 m of plot boundaries and in the same habitats. We categorised our survey locations as “In” plots or “Out” of plots so we could assess potential differences in bird estimates.

Anderson counted birds using four different methods (strip census, strip map, point quarter and sample count methods), but stated that the sample count method produced the most accurate density estimates, which were consistent with his estimates from information combining all his techniques (Anderson 1970, Anderson 1972). The sample count method involved conducting ten 10-minute stationary point counts spaced approximately 95 m apart along an “irregular” transect. We assumed that “irregular” referred to a transect with a non-straight vector, but are unsure if Anderson's transects followed roads and trails or simply transected forest. The forest understorey in his plots is characterised by dense vegetation and steep terrain, conditions that would disturb birds and potentially reduce the number of birds counted, so we hypothesise that his irregular routes followed logging roads and trails to minimise disturbance. At each point, Anderson recorded all birds seen within 18 m and, if a bird was heard, he attempted to visually observe it. He counted birds starting one hour after sunrise at least once per week between 2 November and 1 March 1968 to 1970, on days with good weather.

To maximise our chances of reliably comparing our results with Anderson’s, we also used stationary (point) counts that were spaced approximately 100 m apart along roads and trails and were 10 minutes in duration. Plots were visited four times total, during which we conducted 10 to 15 counts per visit. All counts were conducted from dawn to noon on days with minimal rain or wind. Each plot was surveyed twice each year in January through to March, usually once by each skilled observer (except for plots 3 and 4 which were surveyed twice by the same observer in 2021). The total effort amounted to 35 to 55 total counts in each plot. Numbers of points varied amongst years and plots and are preserved in the data archived here. We made no attempt to repeat surveys at the same locations across years. All survey locatons are identified by their latitude and longitude (see below). During each count we recorded each bird that was detected, the distance to it (checked with laser rangefinder), the cardinal direction of its initial detection and detection type (singing, calling, visual, flyover, drumming). If a bird was heard within approximately 20 m, but was not seen, an effort was made to visually locate that individual. Thus, we repeated Anderson’s methods as closely as possible. At the same time, we used modern counting methods with unlimited radius count areas and time-interval tracking of individual birds where each count was divided into ten 1-minute intervals (or in a few cases two 5-minute intervals). We also measured the latitude and longitude of each point location with hand-held GPS units, typically Garmin etrex 10 (accurate to within 10 m).

Counts were conducted as back-to-back 5-min surveys totalling 10 min each. Within each 5-min count, each bird was given a detection-no detection (dnd) history, based on whether or not it was detected in each of the five one-minute intervals. A 1 indicates the bird was detected (irrespective of detection cue type) within that interval. A 0 indicates it was not detected. Thus, dnd histories are strings of five 0 s and 1 s with one entry of a 1 or 0 per interval. An exception was for 1455 records where the dnd histories included just two total intervals for a 10-min count. In the latter case, if a bird was detected during the first 5 min of the 10 min count, it was noted as 1 in the min1 interval and, if it was not detected, it was noted as 0 in the min1 interval. Likewise, for the second 5-min of a 10-min count, if a bird was detected, it was noted as 1 in the min2 interval and 0 if it was not.

**Habitat surveys**

Anderson recorded trees per acre, average canopy cover, height class distribution, shrub density and qualitative descriptions of vegetation in each plot. Anderson used a variety of methods to produce his results. We used methods described by James and Shugart (1970) to produce the same metrics. We spaced vegetation plots at intervals of approximately 100 m along the same roads and trails where we surveyed birds. The centre of each circular plot of 12-m radius was located 15 to 20 m perpendicular to the trail or road. We identified the species and diameter at breast height category of each tree, measured maximum canopy height with laser rangefinders and counted the number of shrubs over 1 m tall in a 2-metre-wide swath along north-south and east-west transects through the diameter of the plot.

## Geographic coverage

### Description

We surveyed avian communities and vegetation structure at seven of Anderson's original 10 plots in the eastern foothills of the Coast Mountain Range outside of Corvallis, Oregon, USA. These plots are described below using their quarter-section and the degrees-decimal latitude and longitude coordinates of each corner (Tables 1, 2).

## Taxonomic coverage

### Taxa included

**Table:** 

Rank	Scientific Name	
kingdom	Animalia	
phylum	Chordata	
class	Aves	birds
kingdom	Plantae	
phylum	Coniferophyta	conifers
phylum	Magnoliophyta	flowering plants

## Temporal coverage

**Data range:** 2019-6-01 – 2022-2-28; 2022-3-01 – 2022-4-30.

### Notes

Bird surveys were conducted between December 2019 and February 2022. Vegetation surveys were conducted between March and April 2022.

## Usage licence

### Usage licence

Creative Commons Public Domain Waiver (CC-Zero)

## Data resources

### Data package title

Survey of winter bird communities and vegetation in the Oregon Coast Range

### Resource link

https://doi.org/10.5061/dryad.rxwdbrvc8

### Number of data sets

2

### Data set 1.

#### Data set name

Clements_Robinson_winterbirdsurveys_OregonCoastRange.csv

#### Data format

.csv

#### Download URL

https://doi.org/10.5061/dryad.rxwdbrvc8

**Data set 1.** 

Column label	Column description
English Name	Contains the English species name of each bird species following the American Ornithological Society 2021 species’ names (http://checklist.americanornithology.org). R. T. Chesser, S. M. Billerman, K. J. Burns, C. Cicero, J. L. Dunn, B. E. Hernández-Baños, A. W. Kratter, I. J. Lovette, N. A. Mason, P. C. Rasmussen, J. V. Remsen, Jr., D. F. Stotz and K. Winker. 2021. Check-list of North American Birds (online). American Ornithological Society. When no birds were detected during a count, passerine sp was used and zero was entered into all count interval bins. One subspecies was noted: Yellow-rumped Warbler (Myrtle).
Scientific Name	Contains the scientific species name of each bird species following the American Ornithological Society 2021 species’ names (http://checklist.americanornithology.org). R. T. Chesser, S. M. Billerman, K. J. Burns, C. Cicero, J. L. Dunn, B. E. Hernández-Baños, A. W. Kratter, I. J. Lovette, N. A. Mason, P. C. Rasmussen, J. V. Remsen, Jr., D. F. Stotz and K. Winker. 2021. Check-list of North American Birds (online). American Ornithological Society. When no birds were detected during a count, passerine sp was used and zero was entered into all count interval bins.
Direction	To help observers keep track of different individual birds detected within a survey, direction was noted, based on the location of each bird at its initial detection. East=e, West=w, North=n, South=s, North-northeast=nne, Northeast=ne, East-southeast=ese, Southeast=se, South-southwest=ssw, Southwest=sw, West-northwest=wnw, Northwest=nw, North-northwest=nnw. Direction not noted (cell filled with NA) if no birds were detected (passerine sp entered for English Name).
Distance	Distance between the observer and each bird at its initial point of detection was estimated and measured, when possible, to the nearest 5 m up to 50 m, the nearest 10 m up to 100 m and the nearest 25 m at greater distances when possible. Detections within 20 m were noted to within 1 m if the bird was seen. A few records above 20 m were also measured more precisely if the bird was seen or if circumstances allowed more precise measurements, such as recognition a bird must be in a specific tree. Distance not noted (cell filled with NA) if no birds were detected (passerine sp entered for English Name).
cfsvd	Detection cues. The cues by which each bird was detected are noted, including calls (c) , song (s), drumming by woodpeckers (d), visuals (v) and fly-over (f). More than one cue can be noted for any given bird.
min1	Noted as 1 if a bird was detected and 0 if it was not during the first minute of a 5-min count.
min2	Noted as 1 if a bird was detected and 0 if it was not during the second minute of a 5-min count.
min3	Noted as 1 if a bird was detected and 0 if it was not during the third minute of a 5-min count.
min4	Noted as 1 if a bird was detected and 0 if it was not during the fourth minute of a 5-min count.
min5	Noted as 1 if a bird was detected and 0 if it was not during the fifth minute of a 5-min count.
Time	Start time of each 5-min count period.
Lat	Latitude in decimal degrees. Measured to 10 m accuracy with hand-held GPS unit.
Long	Longitude in decimal degrees. Measured to 10 m accuracy with hand-held GPS unit.
Plot	Anderson plot within which survey stations were located. Each survey location is noted with latitude and longitude coordinates. Each location is the plot where stationary (point) counts were surveyed. Plots begin with the abbreviation AP (Anderson Plot) followed by a number between 3 and 9 because we included Anderson’s original plots that he numbered 1-10. We excluded plots 1, 2 and 10 because of radical changes in habitat composition since his original surveys (usually clear-cutting) or dominance by oaks instead of coniferous trees.
CountLoc	CountLoc. Survey point locations with plots and whether they were within the quarter-sections originally designated by Anderson as his plots or not. In = latitude and longitude coordinates place the survey location within his original plot. Out = latitude and longitude coordinates place the survey location outside his original plot, but within 250 m of its boundary and containing habitat that, when viewed on satellite imagery taken within a year of our surveys, had similar habitat to that in the nearby plot. Exclude = latitude and longitude coordinates of surveys we conducted that were more than 250 m outside the boundaries of Anderson’s original plots or within 250 m, but in habitat that was substantially different from that in 1970.
Date	DD/MM/YYYY on which each bird was detected.
obs	Observer who conducted the count. Nolan Clements (nmc), W. Douglas Robinson (wdr).
notes	An additional notation indicating that 1455 records used two 5-min time intervals instead five 1-min intervals in back-to-back 5-min counts is made.
Crediting data use	A reminder is entered: If using any record(s) from this dataset in electronic or other published works, including other datasets, the record(s) are to be fully cited as: Clements, N.C. and Robinson, W. D. 2022. A re-survey of winter bird communities in the Oregon Coast Range, USA, initially surveyed in 1968-1970. Biodiversity Data Journal. (Once accepted and published, the full citation will be entered here).

### Data set 2.

#### Data set name

Clements_Robinson_vegetationsurveys_OregonCoastRange.csv

#### Data format

.csv

#### Download URL

https://doi.org/10.5061/dryad.rxwdbrvc8

**Data set 2.** 

Column label	Column description
Date	DD/MM/YYYY vegetation measurements were made in the field.
Plot	Anderson plot within which survey stations were located. Each survey location is noted with latitude and longitude coordinates. Each location is the plot where stationary (point) counts were surveyed. Plots begin with the abbreviation AP (Anderson Plot) followed by a number between 3 and 9 because we included Anderson’s original plots that he numbered 1-10. We excluded plots 1, 2 and 10 because of radical changes in habitat composition since his original surveys (usually clear-cutting) or dominance by oaks instead of coniferous trees.
Lat	Latitude in degrees decimal. Measured to 10 m accuracy with hand-held GPS unit.
Long	Longitude in degrees decimal. Measured to 10 m accuracy with hand-held GPS unit.
Total Shrub	Count of the total numbers of shrubs encountered in two perpendicular transects (aligned north-south and east-west) that spanned the diameter of the circular plots, following details in James and Shugart (1970).
Height	Height in metres of the tallest tree within the circular plot. Measured with a laser rangefinder to the nearest metre.
Total Tree	Count of the total numbers of trees encountered within each circular vegetation plot. Definition of tree follows that described by James and Shugart (1970).
Tree species columns (12)	The next 12 columns are counts of each tree species in each plot. Douglas fir (Pseudotsugamenziesii), big leaf maple (Acer macrophyllum), western hemlock (Tsuga heterophylla), grand fir (Abies grandis), Oregon white oak (Quercus garryana), red alder (Alnus rubra), western red cedar (Thuja plicata), ponderosa pine (Pinus ponderosa), madrone (Arbutus menziesii), Pacific yew (Taxus brevifolia), unidentified conifer and unidentified hardwood. WFO (2022): World Flora Online. Published on the Internet; http://www.worldfloraonline.org
Tree size columns (8)	The last eight columns are the counts of trees within diameter at breast height categories according to the criteria of James and Shugart (1970). Categories are labelled as a letter from A (smallest) to H (largest). We added the dbh values in inches and centimetres behind each column letter label according to measurements in James and Shugart (1970).

## Figures and Tables

**Table 1.** Size and location of each plot, based on township, range, section and quarter-section in Benton County, Oregon, USA.

Plot Number	Township	Range	Section	Quarter
3	11S	5W	9	NW
4	11S	5W	7	SW
5	11S	5W	8	SW
6	12S	7W	15	NW
7	12S	7W	10	SE
8	11S	5W	8	SE
9	12S	7W	16	NW

**Table 2.** Location of each plot based on degrees-decimal latitude and longitude coordinates of each corner.

	**Northwest corner**		**Northeast corner**		**Southwest corner**		**Southeast corner**	
Plot	Latitude	Longitude	Latitude	Longitude	Latitude	Longitude	Latitude	Longitude
3	44.63582	-123.31238	44.63582	-123.30209	44.62921	-123.31238	44.62921	-123.30209
4	44.62921	-123.35327	44.62921	-123.34378	44.62118	-123.35327	44.62118	-123.34378
5	44.62921	-123.33279	44.62921	-123.32346	44.62118	-123.33279	44.62118	-123.32346
6	44.53446	-123.53619	44.53446	-123.52638	44.52732	-123.53619	44.52732	-123.52638
7	44.54191	-123.52638	44.54191	-123.51644	44.53446	-123.52638	44.53446	-123.51644
8	44.62921	-123.32346	44.62921	-123.31238	44.62118	-123.32346	44.62118	-123.31238
9	44.53446	-123.55616	44.53446	-123.54663	44.52732	-123.55616	44.52732	-123.54663
